# Hearing impairment in MELAS: new prospective in clinical use of microRNA, a systematic review

**DOI:** 10.1186/s13023-018-0770-1

**Published:** 2018-02-21

**Authors:** Arianna Di Stadio, Valentina Pegoraro, Laura Giaretta, Laura Dipietro, Roberta Marozzo, Corrado Angelini

**Affiliations:** 1 San Camillo Hospital IRCCS, Via Alberoni, 70 Venice, Italy; 2 Highland Instruments, Cambridge, MA USA

**Keywords:** MELAS, Hearing loss, Hearing impairment, Otoacustic emission, Auditory brain response, microRNA, Diagnosis

## Abstract

**Aim:**

To evaluate the feasibility of microRNAs (miR) in clinical use to fill in the gap of current methodology commonly used to test hearing impairment in MELAS patients.

**Material and method:**

A literature review was performed using the following keywords, i.e., MELAS, Hearing Loss, Hearing Impairment, Temporal Bone, Otoacustic Emission (OTOAE), Auditory Brain Response (ABR), and microRNA. We reviewed the literature and focused on the aspect of the temporal bone, the results of electrophysiological tests in human clinical studies, and the use of miR for detecting lesions in the cochlea in patients with MELAS.

**Results:**

In patients with MELAS, Spiral Ganglions (SG), stria vascularis (SV), and hair cells are damaged, and these damages affect in different ways various structures of the temporal bone. The function of these cells is typically investigated using OTOAE and ABR, but in patients with MELAS these tests provide inconsistent results, since OTOAE response is absent and ABR is normal. The normal ABR responses are unexpected given the SG loss in the temporal bone.

Recent studies in humans and animals have shown that miRs, and in particular miRs 34a, 29b, 76, 96, and 431, can detect damage in the cells of the cochlea with high sensitivity. Studies that focus on the temporal bone aspects have reported that miRs increase is correlated with the death of specific cells of the inner ear.

MiR − 9/9* was identified as a biomarker of human brain damage, miRs levels increase might be related to damage in the central auditory pathways and these increased levels could identify the damage with higher sensitivity and several months before than electrophysiological testing.

**Conclusion:**

We suggest that due to their accuracy and sensitivity, miRs might help monitor the progression of SNHL in patients with MELAS.

## Background

MELAS, an acronym for myopathy, encephalopathy, lactic acidosis and stroke like episode syndrome [1], is a mitochondrial disease that can arise from 10 different mitochondrial DNA (mtDNA) mutations; in 80% of the cases it is caused by a 3243A > G point mutation in the leucine transfer RNA gene [1, 2]. The prevalence of the 3243A > G mutation in Caucasian population it has been recently re-evaluated and authors identified a 0.24% of prevalence (236/ 1 00000) [3]. This mutation determines an alteration in the protein production by mRNA and change in complexes involved in the respiratory chain (I and IV) [1]. The alteration in metabolism deriving from the mitochondrial pathology leads to a multi-organ disease that involves the ear, as well as muscle, brain, heart, and pancreas [2].

Hearing disorders in MELAS are progressive and related to the severity of the mitochondrial disorder [4]. A large, multi-center study published in 2014 reported that up to 58% patients with MELAS suffer from SNHL [2], whose severity can vary from mild to severe-profound hearing loss [5, 6] depending on the severity of the underlying mitochondrial disorder. Among mitochondrial disorders, MELAS shows the highest incidence of hearing loss [2].

**Fig. 1:**
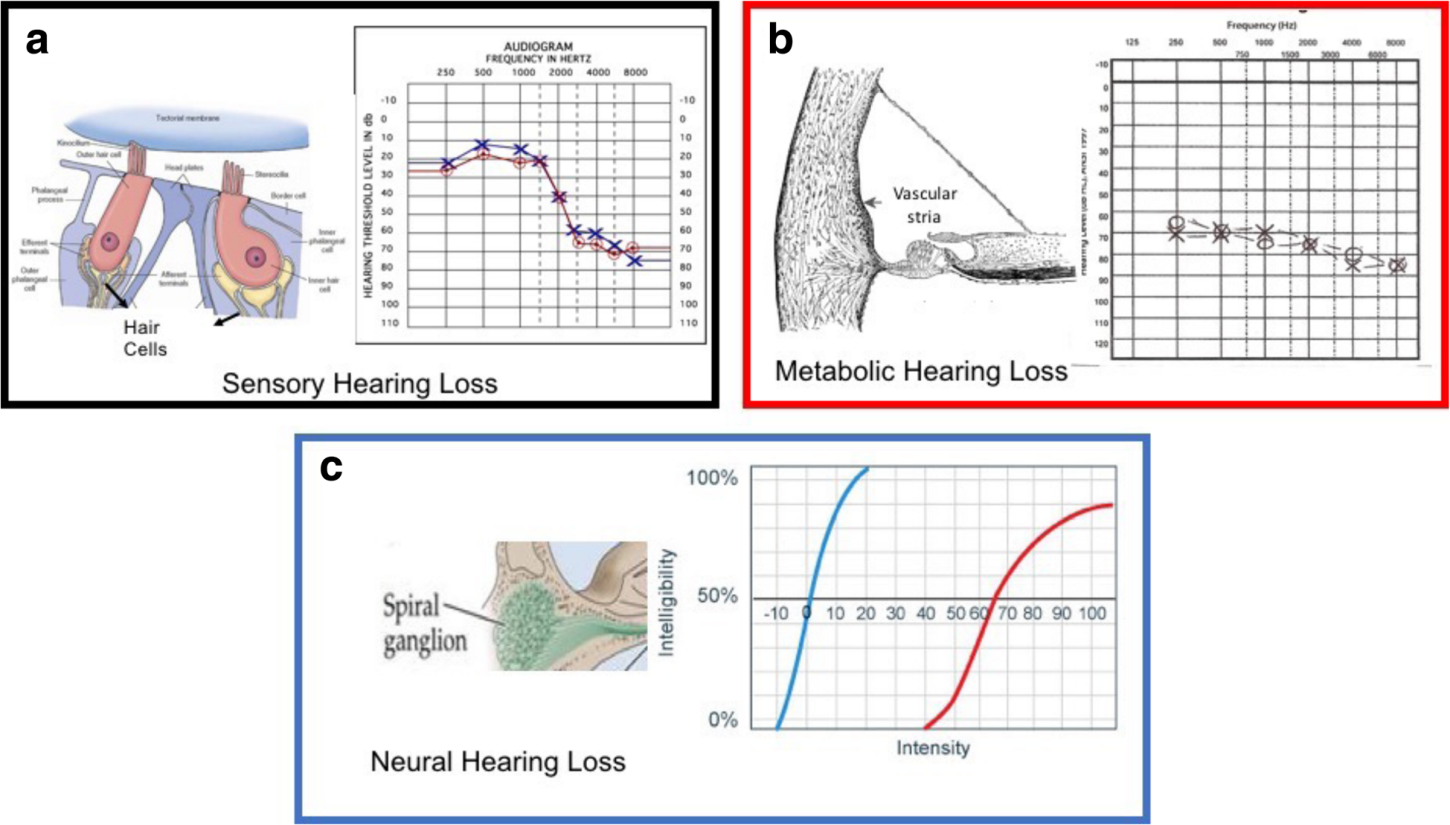
The image shows the different shapes as a function of inner ear cells damage as recorded during a pure auditory test. **a** SensoryNeural Hearing Loss; **b** Metabolic hearing Loss; **c** Neural Hearing Loss

**Fig. 2:**
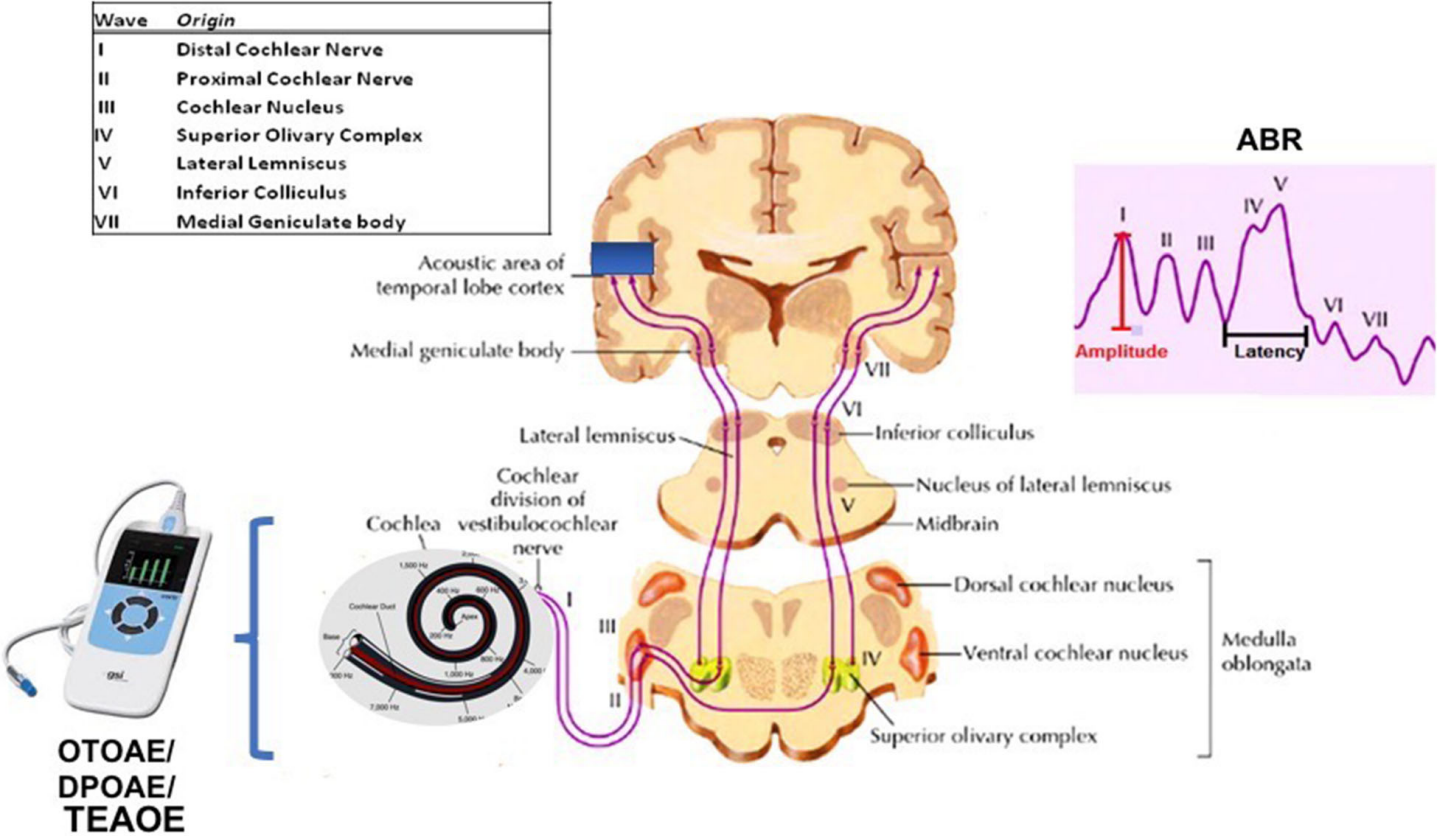
Two methods are used for investigating the hearing pathways. Cochlear function is investigated by OTOAE/TEOAE/DPOAE (which can identify the hair cells damage). The retro-cochlear portion is investigated by ABR. The table on the left shows the area that generates the specific wave; the image shows the area and the wave

**Fig. 3:**
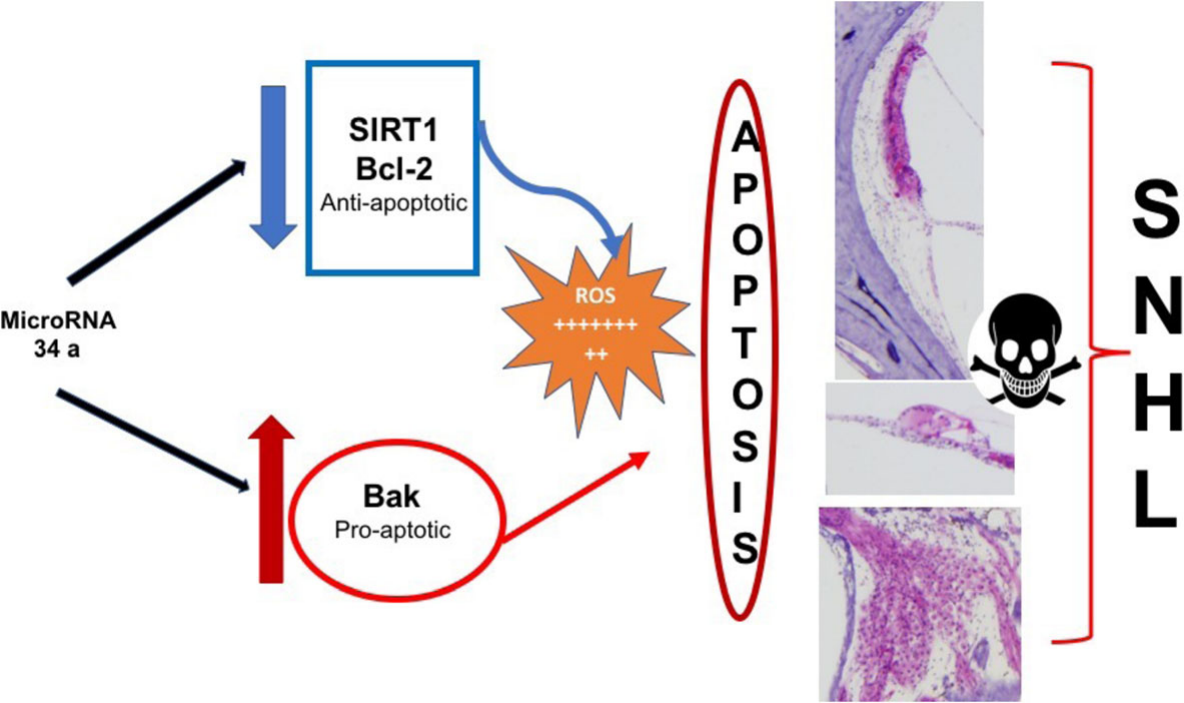
The image summarizes the mechanisms regulated by miR 34 a able to determine the damage of the inner ear structures. From top to bottom: Stria Vascularis, Organ of Corti with hair cells, and Spiral Ganglions in the human temporal bone

Schucknect and Gacek described four forms of Sensorineural Hearing Loss (SNHL) [7, 8], namely: 1) ***Sensory*** when hair cells are the most affected; this SNHL form is characterized by a down-sloping audiogram (Fig. 1a); 2) ***Neural*** when SGs are the most damaged structures; this SNHL form is characterized by a stable pure tone threshold and a progressive loss of word discrimination (Fig. 1b); 3) ***Metabolic*** when SV is the most affected structure; this SNHL form shows a flat or slightly descending pure-tone threshold with good word discrimination (Fig. 1c); and 4) ***Cochlear Conductive*** when structures different from the ones described in the other three forms are the origin of SNHL; this SNHL form is characterized by a gentle down-sloping threshold [6–8]. Sensory, neural and metabolic forms of SNHL (and their typical auditory thresholds) can be caused by a mitochondrial disease, since mitochondria are present in all types of inner ear cells, however they are not homogeneously distributed due to the stochastic segregation; the conductive form cannot be symptom of MELAS because the structure prevalently affected is the middle ear- bone part-.

In clinical practice, the progression of SNHL in patients with MELAS is monitored using a simple pure auditory test (PTA). The auditory threshold shapes recorded during the PTA change depending on which cells are affected by degeneration. However, due to the high variability of auditory thresholds in patients with MELAS [5, 6], this test cannot identify which specific structure of the cochlea is damaged.

Electrophysiological tests are used in clinical practice to improve the efficacy of PTA. While they can detect damage in the cochlea and neural structures, it has been shown that they cannot evaluate which specific cells of the inner ear are damaged [5].

A recent animal study by Prasad et al. [9] has shown that miRs can detect degeneration of the inner ear cells with considerable specificity, but whether miRs is able to detect and characterize hearing impairment in humans has not been thoroughly investigated yet. This review presents the state-of-the-art on the use of miRs for SNHL monitoring; we focused our review on patients with MELAS, where the origin of SNHL in this disease is well understood.

## Main test

### Materials and methods

We conducted a literature search on PubMed, Scopus and Google Scholar using the following keywords: “MELAS, Hearing loss, Hearing Impairment, Temporal Bone, Otoacustic Emission (OTOAE), Auditory Brain Answer (ABR), and microRNA (miR)”. A total of 250 articles were found.

After screening their abstracts, 38 papers were read entirely, 15 were excluded because not relevant to this study, and 32 were selected to be included in our review.

## Results

### Temporal bone aspect and mitochondrial alteration in patients with MELAS

In patients with MELAS, SV displays severe atrophy that affects all turns of the cochlea [10]; additionally, the SGs are reduced in number when compared with SGs in gender- and age-matched healthy subjects [10, 11]. The residual SV cells show vacuole formation and small dark cells which are normally not present in the structure; residual SGs are affected by several degenerative processes such as loss of cell membrane outline and loss of nuclear definition [12].

Takahashi et al. [10] reported that Organ of Corti showed no alterations, and that inner and outer hair cells were normal in number and function; however, these findings could be due to the fact that in this study patients were under 30 years old; unfortunately, other MELAS temporal bone studies descriptions are scarce.

The greater involvement of SV and SGs compared to hair cells might be due to the fact that in these structures the concentration of mitochondria is higher than in hair cells [13].

Mitochondrial mutations in the temporal bone have been studied, but the reported rates are inconsistent across studies. Takahashi et al. reported that SV and Organ of Corti were the most affected structures, with a load of mutations between 78% and 85%, respectively [10].

Koda et al. [11], instead, reported a higher mutation load in SGs than in hair cells and SV and this is partially consistent with data observed in human temporal bone, where SV is more affected by damage than SGs. This is consistent, in part, with the findings reported by Takahashi et al. [10], who in the Organ of Corti observed a mutation load (indicative of mitochondria disorders) higher than in the SV.

We suggest that these inconsistencies can be explained by mitotic segregation. The random distribution of mitochondria at the time of cell division modifies the distribution of mitochondria [14]; thus, the temporal bone changes can be very different among patients affected by MELAS mutation, which might explain the different phenotypes.

Both MELAS mutation or mtDNA deletion in mitochondria modify the production of cytochrome oxidase complex IV. It has been shown that the resulting biochemical deficit of cytochrome although not directly responsible for SG and SV loss, is directly correlated with an increase of Reactive Oxigens Species (ROS) production [15] that induces damage in different parts of the cochlea. The ROS can act on different structures of the cochlea thereby damaging SV, SGs and hair cells, which could further explain the lack of consistency observed in the temporal bone studies [16].

### Audiological findings

The auditory tests of patients with MELAS that have been analyzed in temporal bone studies present flat and down sloping curves always associated with altered word discrimination. The auditory tests show a progression in SNHL correlated with time since mitochondrial disease onset carries a direct relationship with the aggressiveness of the pathology [17–20]. The down-ward sloping curve is observed even when the number of cells of the Organ of Corti is preserved [12]. This can be explained by a reduced function of the hair cells, probably related to the same degeneration observed in the residual SV and SGs.

SNHL in patients with MELAS is commonly bilateral [5, 6, 19, 20]; the unilateral form is present only in 2% of cases [4, 17]. In both forms, SNHL affects high frequency in the onset (75%) and, then involves mild and low frequency [4–6, 17, 19, 20]; in the remaining 25% of case SNHL affects all frequencies in its onset [4, 17].

In clinical studies, patients with MELAS are evaluated, in addition to PTA, with a number of other tests, including Transient Evoked Otoacustic Emission (TEOAE) [4], Otoacustic Emission (OTOAE) [4, 17], Auditory Brain Response (ABR) [4, 5, 17], Psycoacusting Tuning Curves (PTC) [5], Distortion Product Otoacustic Emission (DPOAE) [6, 17], electrocochleography [6, 17], and electrically-evoked compound action potentials [5].

Zwirner et al. [4] observed that MELAS patients suffered from a mild form of SNHL affecting the high frequencies [18] and from moderate to severe SNHL forms involving all frequencies. The word recognition test score was normal in subjects with mild SNHL and abnormal in subjects with moderate to severe SNHL, with a score depending on the SNHL severity. Patients with SNHL with a loss of 40 dB showed normal OTOAE; those with moderate to severe SNHL presented no OTOAE response. In this study, ABR was recorded using a stimulus that consisted of alternating clicks presented at a rate of 16.7/s and were generated by square-wave electrical pulses of 0.1 milliseconds duration. Stimuli were presented monaurally at 80, 90, and 100 dB normal Hearing Level (nHL). Average values of 2000 trials were obtained on stimulation of each ear. In all patients, ABR was normal in latency and amplitude.

Kullar et al. [5] reported that 8/11 MELAS patients with mutation m3243A > G suffered from SNHL, which ranged from mild/moderate hearing loss in high frequencies (5/11 patients) to severe/profound hearing loss (3/11 subjects) spanning all frequencies. These results can be described in terms of the auditory threshold shapes described above, and summarized by both flat and down-ward sloping curves (Fig. 1). Those patients showed complete absence of TEOAEs in all forms of SNHL, reflecting a completely loss of function in outer hair cells. In this study, ABR was recorded using a click stimulus with alternating polarity delivered at a suitable sensation level to give a clear response. The sensation level was predetermined by the mean hearing level from each ear at 2/4 kHz: 440 dB Hearing Level (HL) used click stimulus at 70 dB nHL, 40–60 dB HL used click stimulus at 80 dB nHL, and 460 dB HL used click stimulus at 90 dB nHL. Contralateral masking was applied when required. In two of the 3 patients with profound SNHL, the ABR was not recordable; in the remaining patients, ABR waves displayed normal latency and amplitude even in patients with SNHL. PTC, which allows functional evaluation of inner and outer hair cells at the same time, showed no tip shifts in patients with normal hearing and mild SNHL; shifts at 1 kHz were observed in patients with moderate to severe SNHL in 66% of cases. The shift at 1 kHz indicates complete loss of inner and outer hair cells in the middle turn of the cochlea (Fig. 2).

Santarelli et al. [6] analyzed data from 10 patients with MELAS. They reported a flat threshold curve in all patients. Only 20% of subjects suffered from severe to profound SNHL, while the remainder of patients showed mild to moderate SNHL. DPOAE were detected in 1 ear in 6 out of 10 (60%) of patients. DPOAE responses were identified only at low frequencies in 3 out of the 6 subjects. The DPOAE testing results indicate that outer hair cells function is preserved in some portion of the cochlea, and in particular in HCs in basal turn. One of the two patients with severe to profound SNHL showed preserved DPOAE in both ears, but the ABR waves were not detectable.

Electrocochleography showed normal results in both ears in terms of potential peak amplitude but, the potential displayed lower amplitude when compared with potentials recorded from patients with normal hearing. In the other MELAS patients (i.e., patients with moderate SNHL) electrocochleography testing showed potentials similar to those recorded from normal hearing subjects in terms of peak amplitude, while the evoked potential was altered and resembled the shape typically recorded from patients with hearing impairment.

Sue et al. [17] analyzed 20 patients with MELAS and found moderate to profound SNHL in 78% of patients. The auditory threshold presented a down-ward curve at the onset of SNHL, which then became flat with the disease progression and aging. Only 50% of patients displayed normal speech recognition, suggesting a good retro-cochlear function. ABR was performed using a rarefaction click stimuli; stimulus intensity was at 65 to 70 dB above hearing thresholds or at maximal stimulator output (110 dB) if the hearing threshold was above 40 dB. ABR showed absent or delayed wave I in one ear at least in 61% of patients, but waves III and V were always present. Electrocochleography was performed in 11 patients and the test result was found to be normal in 64% of patients; ABR was not recordable from two patients and in the last two the click evoked electrocochleogram was broad. DPOAE were not detectable when observed in the range of frequency interested by severe to profound SNHL in 7/11 patients, but responses were present and electrically recordable when the SNHL was within 40 dB.

In the study by Vandana et al. [19], 6 children and 2 adults with MELAS were investigated; 3 out of 8 patients suffered from moderate to severe SNHL; in two cases the SNHL was subclinical; 1 presented a mild SNHL. All patients presented a down-ward sloping curve and OTOAE were absent in 50% of subjects. Auditory evoked potentials were recorded using a standard protocol. Only in 1 patient ABR showed absence of signal, which is indicative of retrocochlear disease.

In a large cohort study, Iwanicka-Pronicka et al. [20] showed that the shapes of PTA were correlated with specific mitochondrial mutations. They observed a down-ward sloping curve in patients with m.1555A > G and a pantonal shape with slight down-ward sloping at the high frequencies in the patients with mutation 3243A > G. Their results were statistically significant (*p* < 0.05). A prevalence of moderate SNHL was observed in m.3243A > G, and the 97% of patients with this mutation had a family history of hearing loss.

Overall, the studies described above show the limitations of the pure tone auditory test and of the electrophysiological tests. A comparative analysis of results highlighted that there are major inconsistencies between the outcome of OTOAE/TEOAE/DPOAE and ABR testing.

The presence of OTOAE/TEOAE waves were reported even for SNHL with threshold higher of 40 dB [4, 5], where absence of response would be expected. Other studies showed absence of OTOAE/TEOAE response only when SNHL is moderate to severe (> 40 dB) [4, 19].

ABR waves follow a similar trend. Some studies described either normal latency and amplitude in presence of moderate to severe SNHL [3, 18], or instead reported absent in mild forms of SNHL [4, 5, 16].

### MicroRNAs

MicroRNAs (miRs) are endogenous, small sequences of non-coding RNA [21], that have been shown to modulate a wide range of biological function. MiRs regulate post transcriptional mRNA expression binding the 3′-untraslated region of the complementary mRNA sequence and acting as gene modulator [22]. Change in their concentration has been observed in several diseases, including inflammation and aging [23]. miR levels increasing are specifically related to the damaged structure [22]. Their levels associated to hearing disorders have been investigated [24–27] using miRs, which, due to their high stability in blood, can be easily identified [25].

We speculate that miR levels can be the expression of damage but at the same time they might influence the mitochondrial metabolism by acting on it; they could downregulate the Sirtuin (SIRT1) action by increasing ROS [24], suppress the function of Blc-2 by increasing the apoptosis in cells [25], or increase the function of Bak by causing cell death through increased apoptosis [26] (Fig. 3), and then they might modulate the expression of specific genes by increasing apoptosis [28]. The increase of miRs 34a, 29b, 76, 96, 183 and 431 have been identified as potential markers of hearing damage in animal studies [21–26]; among them, only miR34a has been validated in humans [27].

In humans, miR34a increase has been found to be correlated with hearing loss in aging. In particular, its concentration in blood is anti-correlated with the scores of Pure Tone Averages testing [24]. miRs s16- 5p, 24-3p, and 185-5p were identified in subjects with SNHL exposed to noise and the increase in their levels was correlated with the severity of SNHL [23].

miR increase was directly correlated with reduced responses or completely absence of OTOEA; when the hearing damage was electrically identified, the miR 34a and miR-29b levels were also increased in blood [24, 25], showing a specific correlation between the miRs level and the altered response in OTOAE.

**Fig. 4:**
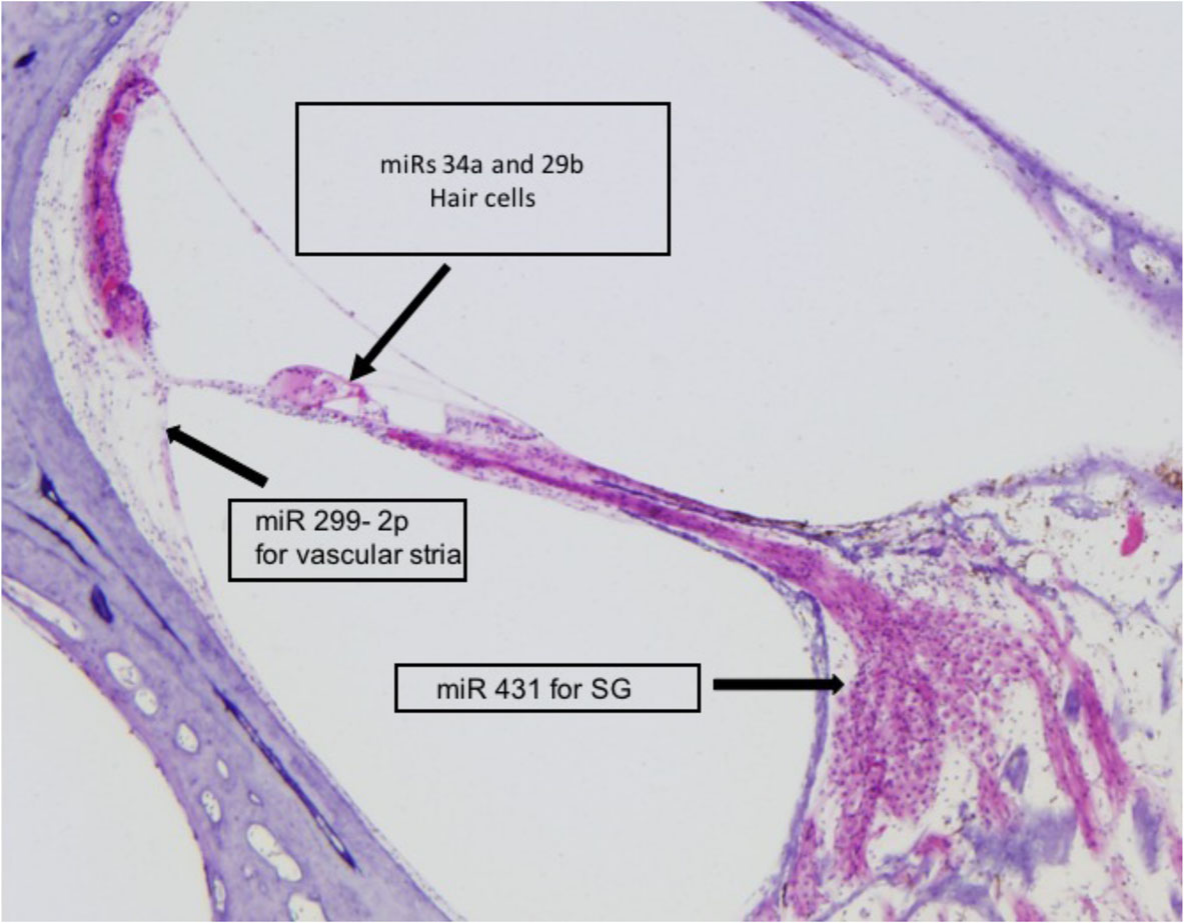
The image shows the details on miR and the structure of the inner ear that,when damaged, can change their levels in blood of patients

The miRs that express cochlear damage are very specific for each structure as shown in Fig. 4, but so far only miRs sensitive to general damage (miR 76) or to hair cells and/or SG damage 34a 96 have been tested in humans.

Jong et al. identified the role of miR-299-3p in the aging vessel process [29] but, until today, nobody use it to evaluate the function of stria vascularis both in animal or human studies; we think that it could be useful due to similar cells present in both structures (vessel and SV).

Meseguer et al. [30] showed the power of miR − 9/9*as a detector of brain damage in humans. Using cybrids from two patients affected from mutation 3243A > G and m8344 A > G, they found that the overexpression of this small molecule was able to increase the mitochondrial dysfunction in MELAS and at the same time provide a measure of brain degeneration. We think that this miR could be helpful to investigate the SNHL in the retrocochlear portion due to its possible increased level when a brain degeneration is ongoing.

In conclusion, the studies described above suggest that miRs can help identify the cells involved in SNHL. Change in miR levels is the expression of cells damage but at the same time miRs can directly modulate the mitochondria metabolism by increasing apoptosis.

## Discussion

Our review identifies the limitations of current clinical method used to evaluate SNHL in MELAS due to the incongruences with the temporal aspect. We suggest to use miRs that could identify the damage that affects the inner ear cells and central hearing pathways with high sensitivity and specificity.

miRs have been proposed as detector of damage in hair cells as well as SGs and SV, which, according to temporal bone studies, are the most damaged structures when SNHL is present in patients with MELAS [10, 11].

In patients with MELAS, SNHL progression is typically first tested with PTA (which is used for screening purposes) and then with OTOAE/DPOAE/TEOAE and ABR (for a more thorough evaluation).

PTA testing alone is not specific enough to identify which cells of the inner ear are affected by the disease. The low PTA sensitivity is due to the variability of auditory thresholds observed in patients with MELAS (which is related to the severity of the mitochondrial disease and time from disease onset [17–20]) as well as to a limitation of the methodology, namely the operator/ patient bias which can lead to a difference of approximately 5 to 10 dB between the actual hearing ability and recorded response.

OTOAE/DPOAE/TEOAE are valid methods to investigate hair cells function [31] and can provide an estimate of which turn of the cochlea suffers from cells loss [6, 7, 17]; however, they cannot evaluate the state of SV. In fact, DPOAE/TEOAE only assess SV in an indirect fashion [6], by measuring the response of hair cells; unfortunately, this indirect method provides results that are not specific enough so it is unable to quantify the actual severity of SV damage. SV (the vascular apparatus of the cochlea) is present in any turn of the cochlea; its atrophy is measured as percentage of “lost area”. When metabolic SNHL occurs, at least 30% of VS is lost, which leads to considerable VS damage [1]. With time, this damage causes hair cells death. The indirect evaluation of SV by DPOAE/TEOAE can delay the diagnosis and lead to an underestimation of the actual damage.

Another limitation of OTOAE/DPOAE/TEOAE is due to the fact that these tests can identify cells damage only when SNHL threshold is over 40 dB; thus, they cannot detect mild SNHL. The identification of SNHL onset (and thus of mild forms of SNHL) is extremely important in MELAS because the use of antioxidant molecules might slow down the progression of the disease and stimulate recovery [4].

SG function is investigated with the word recognition (WR) test, typically followed by the ABR. WR cannot inform on SGs’ damage for two reasons. First, a loss of at least 70% of SGs is necessary to lead to a reduction in the percentage of WR that is classifiable as abnormal [6]; second, brain structures (which are necessary for speech recognition [32]) could compensate for the reduction of SGs, especially if brain function is fully preserved.

ABR allows to investigate the hearing pathway from SG to the auditory cortex. Each wave recorded as a response to the test assesses the function of a specific area (Fig. 2). A damage in a specific area of the hearing pathways is reflected by a change in ABR amplitude and/or latency.

Normal ABR waves have been found in patients with MELAS [5, 6]. This finding is not consistent with the data reported in temporal bone studies [5, 6, 8, 10, 15] that have shown that SGs are reduced in number or present anatomical abnormalities inconsistent with normal function, as well as with the damage caused by stroke in MELAS. Instead, in patients with MELAS we expect to see abnormal ABR latencies, at least. Normal ABR waves are not consistent with clinical observations and the findings observed in human temporal bone.

The inconsistencies are probably due to several causes. A key cause is probably the low specificity of waves I in identifying which structures are affected by damage. Waves I record the electrical potentials associated with the global activity of hair cells synapsis, SGs and cochlear nerve; thus, isolating the contribution of the SG damage alone is difficult. This difficulty is compounded by signal amplification by the cochlear nerve. After the cochlear nerve, the signal travels upward and reaches the cochlear nucleus (wave III), the superior olivar complex (wave IV) and lateral lemniscus (wave V). The damage due to a stroke or metabolic dysfunction needs to involve a wide area of the auditory pathways in order to determine a change in the ABR response, similar to what happens with ABR response and SG damage.

The limitations of the electrophysiological tests described above highlight the need of identifying an alternative, more specific method to investigate the hearing pathways. This could be especially beneficial for patients, like MELAS patients, where the progression of the hearing impairment can be slowed down via pharmacological (for example antioxidant) therapy if diagnosis is made early.

MiRs have been shown to be very specific and highly sensitive to identify cellular damage in the cells of the inner ear [24–27], vascular structure [29] and superior hearing pathways [30].

The validity of miRs 34a and 29b as identifiers of hair cells and SG damage is supported by human studies that identified also the presence of miRs s16- 5p, 24-3p, and 185-5p in subjects exposed to noise and exhibiting SNHL without specific correlation with damaged inner ear structure.

miR-299-3p was identified in humans and correlated to vascular degeneration [29]; we speculate that this miR can help evaluate SV degeneration in SNHL. In fact, SV (the vascular apparatus of the inner ear) contains cells similar to the ones in other vessels and damage in this structure could be identified by the same miR that is found to be increased in aging vessels.

MiR 431 has been shown to be sensitive to the decrease of SGs; thus it could potentially be used to increase the specificity of miR 34a and 29b.

## Conclusion

Our literature review suggests that the levels of mRNAs 34a, 29b, 299-3p and 431 might be used to measure inner ear degeneration (Table 1).

**Table 1:** Summary of miR as detector of cells damage in the hearing pathways

Micro RNA	Structure damaged
*miR 29 b* [23]	Hair Cell and Spiral Ganglion
*miR 34 a* [24, 26]	Hair Cell and Spiral Ganglion
*miR 431* [20]	Spiral Ganglion
*miR 299-3p* [28]	Stria Vascularis
miR − 9/9* [29]	Central pathways from cochlear nuclei to brain cortex

This measure could potentially identify the origin of SNHL for instance by miR-9/9*, which has already been identified as a marker of brain degeneration in patients with MELAS might be used to identify the damage in central hearing pathways in the retro-cochlear portion due to its increased level occurring during a process of brain degeneration.

Thanks to their sensitivity and quick response to the change in cells conditions we speculate that miRs might help to assess the effect of antioxidant pharmacological therapy on neural structures. The validity of circulating miR to identify the subtype of Amyotrophic Lateral Sclerosis was previously demonstrated by our group [33].

We have focused our review on patients with MELAS, because in such pathology SNHL and mitochondrial alteration are intimately related.

MELAS patients -where the damage is related to a mitochondrial alteration- can serve as a model to investigate the accuracy of miRs in identifying which cells are damaged, since we infer that the most affected cells are the ones with higher concentrations of mitochondria (SV and SGs). The validity of miRs measurements after confirmed by this model could be applied to evaluate the damage in other forms of SNHL.

Our literature review suggests that miRs might be used to detect damage in the hearing pathways of MELAS patients, especially early on in the disease when SNHL starts manifesting. If early detected, SNHL might be successfully treated with antioxidants [19].

MiRs can be detected in blood, thus sample collection can be easily performed. While miR analysis might increase the cost of monitoring SNHL, we note that mRNAs analysis in patients with rare diseases is widely accepted.

MiRs 34a, 29b, 299-3p and miR431 and − 9/9* might be particular useful for monitoring SNHL, as they can identify which cells are more affected by mitochondrial degeneration both peripherally (inner ear) and centrally (from nuclei to cortex).

MiRs might be used in conjunction with electrophysiological tests to enhance their efficacy for identifying specific cells damage in MELAS.

Future work should focus on testing more carefully their accuracy in humans and investigating to what extent miRs can be used to evaluate SNHL forms that have a different origin.

## Abbreviations

ABR: Auditory brain response
DPOAE: Distortion product otoacustic Emission
MELAS: Myopathy, encephalopathy, lactic acidosis and stroke like episode syndrome
miR: MicroRNA
OTOAE: OtoAcustic emission
PTA: Pure tone auditory test
SG: Spiral Ganglion
SNHL: SensoriNeural hearing loss
SV: Stria vascularis
TEOAE: Transient Evoked OtoAcustic Emission
